# Gemcitabine and Flurbiprofen Enhance Cytotoxic Effects on Cancer Cell Lines Mediated by Mesenchymal Stem Cells

**DOI:** 10.3390/ijms26136212

**Published:** 2025-06-27

**Authors:** Agata Kawulok, Paulina Borzdziłowska, Magdalena Głowala-Kosińska, Wojciech Fidyk, Andrzej Smagur, Barbara Łasut-Szyszka, Agnieszka Gdowicz-Kłosok, Iwona Mitrus, Marcin Wilkiewicz, Agata Chwieduk, Daria Burdalska, Joanna Korfanty, Sebastian Giebel, Marcin Rojkiewicz, Andrzej Bak, Violetta Kozik

**Affiliations:** 1 Institute of Chemistry, University of Silesia, Szkolna 9, 40-007 Katowice, Poland; agata.kawulok@gliwice.nio.gov.pl (A.K.); marcin.rojkiewicz@us.edu.pl (M.R.); 2 Department of Bone Marrow Transplantation and Oncohematology, Maria Sklodowska-Curie National Research Institute of Oncology, Gliwice Branch, Wybrzeze Armii Krajowej 15, 44-101 Gliwice, Poland; paulina.borzdzilowska@gliwice.nio.gov.pl (P.B.); magdalena.glowala-kosinska@gliwice.nio.gov.pl (M.G.-K.); wojciech.fidyk@gliwice.nio.gov.pl (W.F.); andrzej.smagur@gliwice.nio.gov.pl (A.S.); iwona.mitrus@gliwice.nio.gov.pl (I.M.); marcin.wilkiewicz@gliwice.nio.gov.pl (M.W.); agata.chwieduk@gliwice.nio.gov.pl (A.C.); daria.burdalska@gliwice.nio.gov.pl (D.B.); joanna.korfanty@gliwice.nio.gov.pl (J.K.); sebastian.giebel@gliwice.nio.gov.pl (S.G.); 3 Center for Translational Research and Molecular Biology of Cancer, Maria Skłodowska-Curie National Research Institute of Oncology, Gliwice Branch, Wybrzeze Armii Krajowej 15, 44-101 Gliwice, Poland; barbara.lasut-szyszka@gliwice.nio.gov.pl (B.Ł.-S.); agnieszka.gdowicz-klosok@gliwice.nio.gov.pl (A.G.-K.)

**Keywords:** pancreatic cancer, mesenchymal stem cells, gemcitabine, flurbiprofen

## Abstract

Mesenchymal stem cells (MSCs) have recently shown great promise as potential anticancer drug delivery carriers. MSCs exhibit tropism to inflammatory sites, such as tumor beds, and resistance to chemotherapeutics. The aim of this study was to examine the efficacy of gemcitabine (GEM) conjugated with flurbiprofen (FLU) as a potential agent enhancing the GEM cytotoxic effect. Pancreatic cancer cell lines (PCCs), including PANC-1, AsPC-1, and BxPC-3, were studied meticulously. Moreover, the usefulness of bone-marrow-derived mesenchymal stem cells (BM-MSCs) treated with GEM and FLU, and the conditioned media from above these cells (CM) as elements supporting the in vitro action of GEM, inducing apoptosis, necrosis, and inhibiting the cell cycle, was tested. The results showed that CM-GEM exhibited higher cytotoxicity towards the selected PCCs compared to GEM alone. Furthermore, the obtained data revealed lower sensitivity of these cells to treatment, which promotes the utilization of BM-MSCs as potential drug carriers. Based on the presented findings, it seems that applying FLU in the antiproliferative effect of GEM might be regarded as an effective strategy in the therapy of pancreatic cancer, especially in the inhibition of proliferation and induction of cancer cell death.

## 1. Introduction

Pancreatic ductal adenocarcinoma (PDAC) is a deadly disease with an increasing frequency of occurrence worldwide that potentially might become the second leading cause of cancer deaths [1]. Unfortunately, PDAC is usually diagnosed at an advanced stage, resulting in an adverse prognosis for patients with a rather low 5-year survival rate ranging from 2% to 9% [2]. Typically, surgical resection is the most common form of therapy for PDAC, but, in many cases, it is unfeasible due to disease advancement. On the other hand, combination chemotherapy, including FOLFIRINOX conjugated with gemcitabine and nab-paclitaxel, are the essential pharmaceuticals used in systemic PDAC treatment [3]. Enormous progress in diagnostics, perioperative treatment, radiotherapy techniques, and systemic therapies for PDAC has been observed in the last decades, which has considerably improved treatment efficiency. Still, that progress is insufficient [4]. In fact, pancreatic cancer is poorly vascularized and characterized by a dense collagen stroma that composes an almost impenetrable barrier to chemotherapeutics. The tumor microenvironment elements and interaction with pancreatic stellate cells promote the development of tumor cells [5,6,7]. The complicated structure of PDAC makes effective treatment a challenging task. Hence, the introduction of new methods (or their combinations) to improve therapy efficiency is highly desirable.

One of the promising approaches to targeted anticancer therapy is the application of mesenchymal stem cells (MSCs) as a factor that has a direct impact on tumor development. Due to MSCs’ unique properties, including low immunogenicity, strong immunomodulatory and immunosuppressive abilities, and tropism to inflammation, MSCs have also become good candidates for drug carriers. Numerous studies have shown that MSCs can inhibit tumor growth by silencing angiogenesis, increasing inflammatory cell infiltration, activating apoptosis, and stopping the cell cycle [8,9,10]. It has been observed that MSCs can serve as carriers for delivering substances with anticancer activity to primary tumors, as well as metastases. Regardless of the administration procedure, the transplanted MSCs are rapidly eliminated while maintaining therapeutic effects (e.g. immunomodulation) [11].

Recent studies have shown that MSCs are able to take up gemcitabine (GEM) molecules from the environment using nucleoside transporters (NTs), such as human equilibrative NTs (hENTs) and human concentrative NTs (hCNTs), and effectively release them, thus inhibiting the growth of pancreatic cancer cells. The conducted in vitro/in vivo studies have indicated that the use of MSCs as a drug carrier (e.g., gemcitabine) has enabled a medicine dose to be diminished while maintaining therapeutic efficacy [12,13,14]. Additionally, the therapeutic effect induced by MSC administration also results from the secretion of a wide range of growth factors, cytokines, and extracellular vehicles (EVs), which together contribute to tissue regeneration, as well as the amelioration of inflammatory and immune reactions [9,15].

The use of MSCs as drug carriers faces limitations, primarily due to inefficient transfection, which remains a major challenge [16]. Moreover, MSCs, as living cells, may influence the tumor microenvironment by promoting cancer cell proliferation, migration, and drug resistance. These effects, along with drug absorption capacity, vary depending on MSC origin and culture conditions, highlighting the importance of accounting for their heterogeneity [16]. Despite these concerns, MSCs are still considered promising for clinical applications. However, their use in immunotherapy is debated, as their production must comply with Good Manufacturing Practice (GMP) to maintain immunomodulatory properties [17]. Different expansion methods affect these properties, which led to the development of optimized MSC isolation protocols, ensuring safety and functional stability [18,19].

Three main types of extracellular vehicles (EVs) are distinguished: exosomes, microvesicles, and apoptotic bodies. EVs are lipid-bilayer-bound structures containing bioactive molecules such as RNA, DNA, proteins, and lipids, and are involved in intercellular communication, tissue homeostasis, and immune regulation [20]. MSC-derived exosomes have shown significant therapeutic potential in bone repair by promoting osteogenic differentiation, angiogenesis, and modulating immune responses [21]. Their biogenesis can be ESCRT-dependent or independent, and they reach recipient cells via clathrin-mediated endocytosis, receptor interaction, or membrane fusion [22]. MSCs tend to localize in tumor clusters, where their exosomes exert biological effects depending on their molecular cargo—proteins, nucleic acids, peptides, or drugs. Cargo can be introduced into exosomes passively (e.g., drug incubation) or actively (e.g., transfection, sonication), each with specific advantages and drawbacks. Passive loading is simple but may result in uncontrolled drug amounts, making it necessary to thoroughly understand the mechanisms and kinetics of substance loading into MSC-derived exosomes [23].

Gemcitabine (GEM) is the most commonly used drug in the treatment of pancreatic cancer, and it belongs to the pyrimidine antimetabolites. Due to its similarity to 2′-deoxycytidine, GEM is incorporated into the double helix of DNA, disrupting DNA synthesis and leading to cell death. In fact, GEM is a phase-specific drug acting in the S phase of cell division. As a cytotoxic compound, GEM causes cell damage and/or death while being rapidly metabolized and excreted from the body. Thus, in order to increase the effectiveness of the treatment, patients are recommended to take high doses of GEM, which results in the occurrence of side effects. Unfortunately, many types of cancers, including pancreatic cancer, develop resistance to GEM [24,25].

Flurbiprofen (FLU) is a well-known, nonsteroidal anti-inflammatory drug (NSAID), the action mechanism of which is mainly based on the inhibition of cyclooxygenase (COX) enzymes involved in the synthesis of prostaglandins [26]. The preventive and therapeutic effects of NSAIDs have also been examined in different cancers (e.g., colon, pancreatic, liver, breast, ovarian, and thyroid [27,28,29,30,31,32,33,34,35,36,37,38,39,40]). Apart from the anti-inflammatory properties, NSAIDs have revealed anticancer features such as, among others, the ability to induce apoptosis, inhibit angiogenesis, and enhance the cellular immune response. In practice, the most common therapeutic targets for combating inflammation are COX, NF- kB, cytokines/chemokines, fibroblast (VEGF), and vascular endothelial growth factors (FGF) [41,42,43,44,45,46]. It should be highlighted that COX overexpression has been noticed in various cancers (e.g., pancreatic, breast, lung, and colon) over the past few decades. Moreover, the overexpression of COX stimulates angiogenesis, which is a crucial factor in the occurrence of metastases [42].

The anticancer effects of FLU have been documented in thyroid [47], colon [48], and breast cancer models [49,50]. Consequently, combining GEM with NSAIDs is being explored to enhance cancer therapy efficacy. Such conjugation may boost GEM’s cytotoxicity by reducing tumor-promoting inflammation, offering a promising alternative to monotherapy. GEM-NSAID combinations are expected to act synergistically, improving therapeutic outcomes. For example, aspirin inhibited proliferation and enhanced the cytotoxicity of GEM in GEM-resistant pancreatic cancer cells, such as PANC-1, inducing G1 cell cycle arrest [51]. While aspirin alone does not trigger apoptosis, it inhibits GSK-3β activation and downregulates its targets (cyclin D1, Bcl-2), which are involved in cancer cell survival and chemoresistance. Similar effects were observed in Capan-1 cells. Moreover, low-dose aspirin combined with GEM reduced proliferation and migration, promoted apoptosis and E-cadherin expression, and suppressed vimentin more effectively than GEM alone [52]. This combination also significantly inhibited the PI3K/AKT/mTOR pathway in SW1990 and BxPC-3 cells.

The beneficial role of celecoxib in combination with anticancer drugs (e.g., GEM) has also been noticed during advanced (pre)clinical studies confirming the effectiveness of multi-drug therapy [46,53,54,55]. The combination of GEM-FLU is a novel conjugate. The impact of various NSAIDs, including flurbiprofen, on pancreatic cancer cells (BxPC-3 and PANC-1) has been investigated meticulously [56], revealing that flurbiprofen exhibited the strongest antiproliferative and migratory effects on pancreatic cancer cells, which supports the rationale for choosing flurbiprofen among the available NSAIDs. Moreover, the sensitization of pancreatic cancer cells treated with GEM by flurbiprofen has been described as well.

The fundamental objective of the study was to examine the efficacy of GEM conjugated with FLU as a potential agent enhancing the GEM cytotoxic effect. Therefore, apoptosis and necrosis, as well as the expression of apoptotic proteins in pancreatic cancer cell lines (PCCs), including PANC-1, AsPC-1, and BxPC-3, were studied meticulously. Moreover, the usefulness of bone-marrow-derived mesenchymal stem cells (BM-MSCs) treated with GEM and FLU and the conditioned medium from above these cells (CM) as elements supporting the in vitro action of GEM and FLU, was tested. In particular, we focused on BM-MSCs’ resistance to the GEM and FLU drug combination in the context of the ‘tandem’ antitumor efficacy compared to their anticancer properties alone.

## 2. Results

### 2.1. BM-MSC Phenotyping Characterization

**Figure 1:**
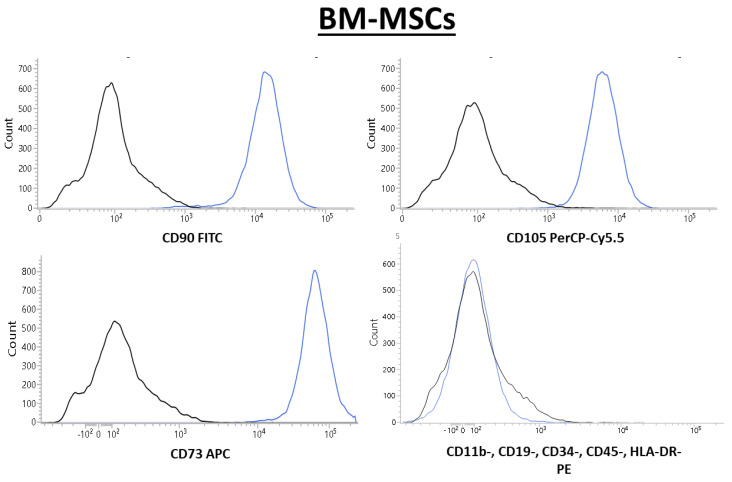
Flow cytometry plots show the presence of surface markers characteristic for MSCs (CD73, CD90, and CD105) and the concomitant absence of blood-cell-lineage-specific markers (CD11b, CD19, CD34, CD45, and HLA-DR). BM-MSCs were stained using the Human MSC Analysis Kit (BD Biosciences). Positive (CD90, CD105, and CD73) and negative (CD11b-, CD19-, CD34-, CD45-, and HLA-DR) antibody cocktails are presented in blue, and the negative isotype control is shown in black.

BM-MSCs isolated from human bone marrow showed normal morphology, adherence to the culture dish, and the ability to proliferate in vitro. Flow cytometric analysis of cells at passage three showed that BM-MSCs had surface markers CD73+, CD90+, and CD105+, but did not indicate the presence of surface markers CD11b-, CD19-, CD34-, CD45-, or HLA-DR-, as presented in Figure 1.

### 2.2. Cytotoxic Effect of GEM + FLU on BM-MSCs and PCCs

**Figure 2:**
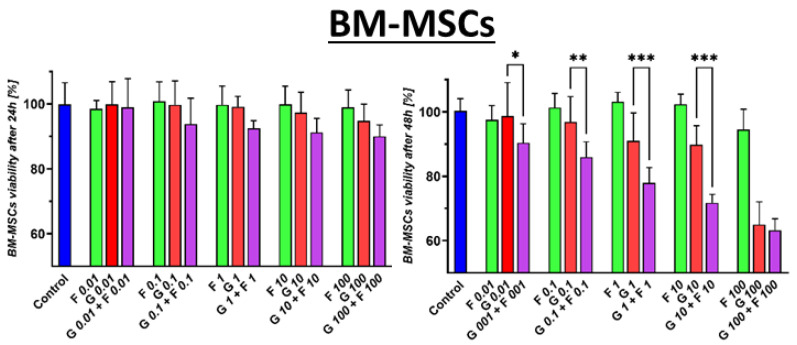
The effect of GEM (red), FLU (green), and GEM + FLU (purple) in equal concentrations (0.01–100 μM) on BM-MSC proliferation was measured by an MTS assay and analyzed using one-way ANOVA, followed by Sidak’s multiple comparisons test, comparing GEM vs. GEM + FLU at each concentration (* *p* ≤ 0.05, ** *p* ≤ 0.01, *** *p* ≤ 0.001). The cells were treated with indicated concentrations for 24 and 48 h. The data are shown as mean ± SD of the triplicate experiment.

After 24 h, BM-MSCs showed a slight sensitivity to GEM in the MTS test, as illustrated in Figure 2. A 10% decrease in cell viability was observed at the highest tested concentration of 100 μM GEM. However, a statistically significant increase in the toxicity of the GEM + FLU combination was demonstrated compared to that of GEM alone. Similarly, the same tendency was observed for a wide range of GEM + FLU concentrations (10 nM–100 μM). FLU alone did not significantly affect cell survival in the 24 or the 48 h test.

**Figure 3:**
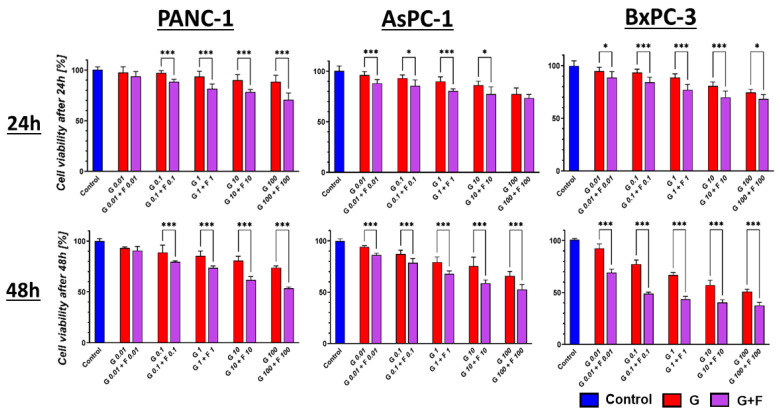
PCC profiling after 24 h and 48 h GEM (red) and GEM + FLU (purple) treatment in equal concentrations (0.01 μM–100 μM). The effect was measured by MTS assay and analyzed using one-way ANOVA, followed by Sidak’s multiple comparisons test, comparing GEM vs. GEM + FLU at each concentration (* *p* ≤ 0.05, ** *p* ≤ 0.01, *** *p* ≤ 0.001). The data are shown as mean ± SD of the triplicate experiments.

Regardless of the concentration of the GEM + FLU combination, the viability of PCC PANC-1, AsPC-1, and BxPC-3 cells was lower than that observed after the use of GEM alone. Differences in the survival of the tested cell lines after GEM and GEM + FLU treatment were visible already at low doses of GEM + FLU (10 nM + 10 nM) after 24 h, as presented in Figure 3.

**Figure 4:**
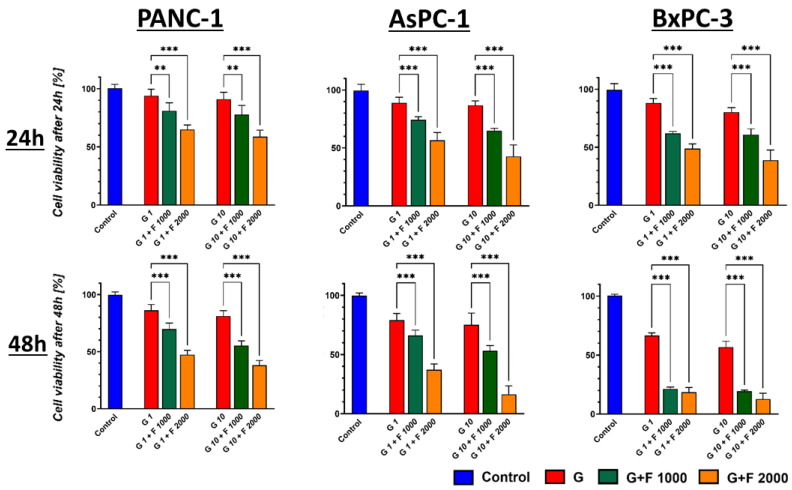
PCC profiling after 24 h and 48 h GEM (red) and GEM + FLU treatment in different concentrations (1 μM + 1 mM (green); 10 μM + 1 mM (green); 1 μM + 2 mM (orange); 10 μM + 2 mM (orange)). The effect was measured by MTS assay. Statistical analysis was performed using Welch’s ANOVA with Brown–Forsythe correction, followed by Dunnett’s T3 multiple comparisons test comparing GEM 1 μM vs. GEM 1 μM + FLU 1 mM/GEM 1 μM + FLU 2 mM and GEM 10 μM vs. GEM 10 μM + FLU 1 mM/GEM 10 μM + FLU 2 mM (* *p* ≤ 0.05, ** *p* ≤ 0.01, *** *p* ≤ 0.001). The data are shown as mean ± SD of the triplicate experiments.

The increment of the FLU dose (1 mM and 2 mM) resulted in a substantial decrease in proliferation with high statistical significance in all tested cell lines, as shown in Figure 4.

The IC_50_ values for GEM and GEM + FLU calculated after 48 h are reported in Table 1.

Compared to GEM, the IC_50_ value for the GEM + FLU combination recorded for the PANC-1 cell line ranged from 109.3 (±9.6) μM to 228.2 (±16.3) μM, while, for BxPC-3, the range was from 1.9 (±0.5) μM to 98.6 (±8.8) μM, indicating an exceptionally high sensitivity of the PANC-1 line to the addition of FLU. In fact, the highest resistance was observed for BM-MSCs, as presented in Table 1.

### 2.3. Cell Cycle Analysis and Morphology of PCCs After GEM + FLU Treatment

**Figure 5:**
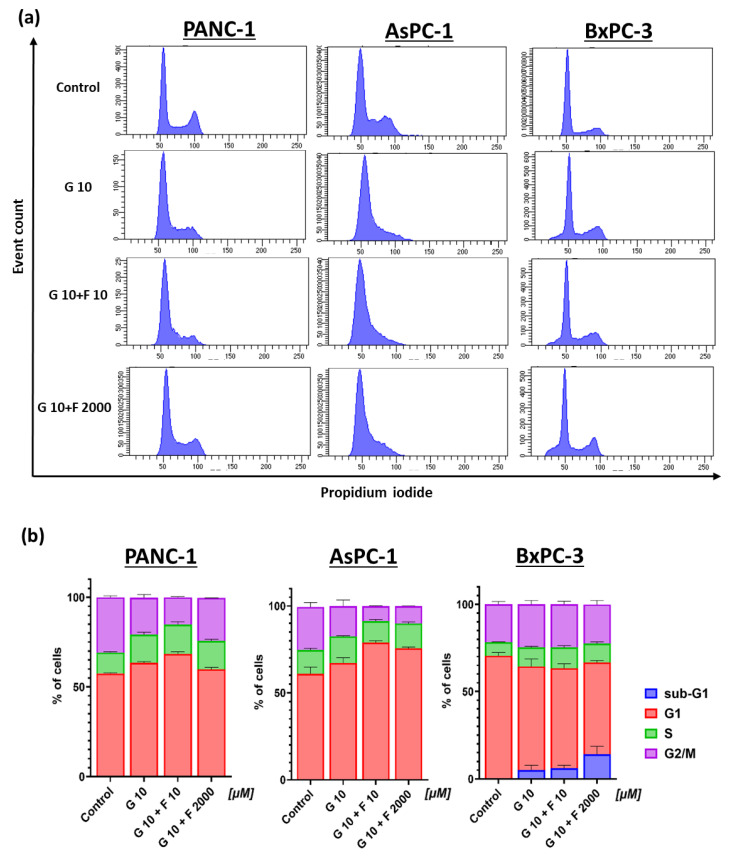
Cell cycle of PCCs after 24 h GEM (10 μM), GEM + FLU (10 μM + 10 μM), and GEM + FLU (10 μM + 2 mM) treatment: (**a**) The dependence of cell number on relative fluorescence intensity for propidium iodide (flow cytometry); (**b**) percentage of cells in the individual phases of the cell cycle (sub-G1 (blue), G1 (red), S (green), and G2/M (purple)).

Cell cycle analysis showed a non-uniform effect of the drugs on individual cell lines. For PANC-1 and AsPC-1 lines, the retention of cell division in the G1 phase was noticed, especially after using the GEM + FLU combination (10 μM + 10 μM) (see Figure 5). Cells in the sub-G1 phase were observed for the BxPC-3 line, particularly after the treatment with GEM + FLU (10 μM + 2 mM), indicating DNA fragmentation, which is characteristic of apoptotic cells.

**Figure 6:**
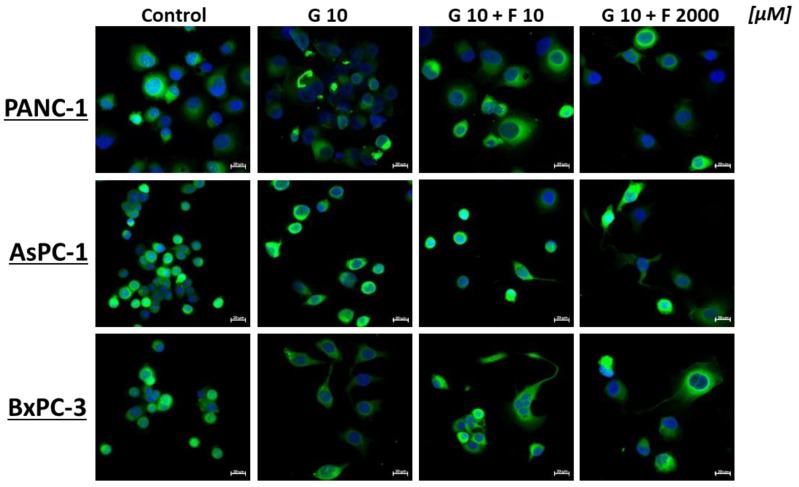
Morphology of PCCs after 24 h GEM (10 μM), GEM + FLU (10 μM +10 μM), and GEM + FLU (10 μM +2 mM) treatment (CFDA staining and DAPI, 40×, Zeiss).

The impact of the investigated molecules resulted in the modification of cell morphology and the vacuolation of PCCs, as illustrated in Figure 6.

### 2.4. Apoptosis and Necrosis of BM-MSCs and PCCs After GEM + FLU Treatment

**Figure 7:**
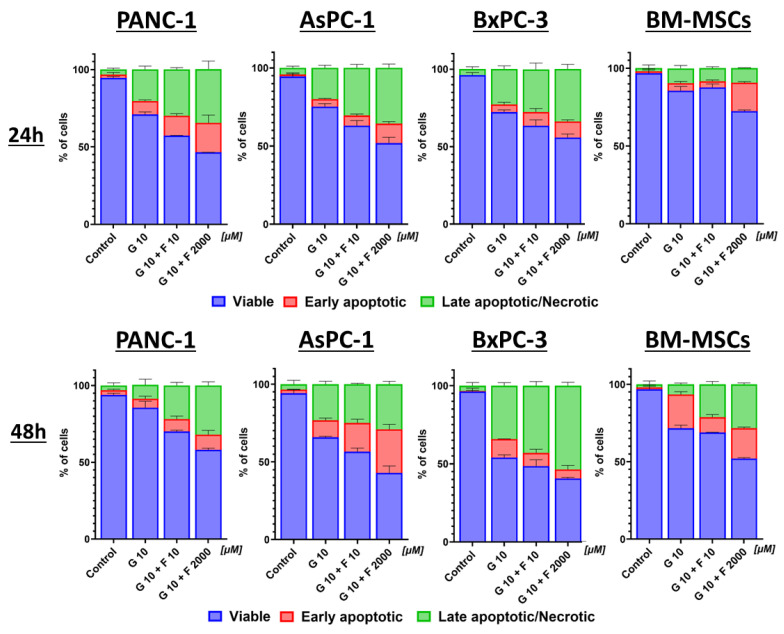
PCC and BM-MSC apoptosis and necrosis after 24 h and 48 h GEM (10 μM), GEM + FLU (10 μM +10 μM), and GEM + FLU (10 μM + 2 mM) treatment (Annexin V and 7-AAD, flow cytometry). The graphs show the percentage of viable (blue), early apoptotic (red), and late apoptotic/necrotic (green) cells after treatment with the appropriate drug combination. Data were analyzed using one-way ANOVA, followed by Sidak’s multiple comparisons test comparing GEM 10 μM vs. GEM 10 μM + FLU 10 μM/GEM 10 μM + FLU 2 mM and GEM 10 μM + FLU 10 μM vs. GEM 10 μM + FLU 2 mM. The data are shown as mean ± SD of the triplicate, independent experiments.

Apoptotic cells in pancreatic cancer cell lines were statistically significantly higher after treatment with the GEM + FLU combination, rather than with GEM alone, especially for high doses of FLU (2 mM), as shown in Figure 7.

In the case of PANC-1, AsPC-1, and BxPC-3 lines, the mean percentage of viable cells after 48 h was 46.4 (±0.1)%, 42.8 (±4.61)%, and 40.5 (±5.78)% for the groups treated with GEM + FLU (10 μM + 2 mM), respectively. The statistical significance of viable PCCs between the groups of GEM (10 μM) vs. GEM + FLU (10 μM + 10 μM), GEM (10 μM) vs. GEM + FLU (10 μM + 2 mM), and GEM + FLU (10 μM + 10 μM) vs. GEM + FLU (10 μM + 2 mM) was *p* < 0.001 after 24 h. In fact, BM-MSCs were characterized by a higher percentage (52.04 ± 0.58%) of viable cells for GEM + FLU (10 μM + 2 mM); however, this was too low to use these cells as carriers of these drugs, therefore, the above combination was excluded from further study. On the other hand, the analysis of the GEM + FLU (10 μM + 10 μM) treatment after 24 h revealed the viability of the treated group at the level of 87.68(± 2.15)%. The statistical significance of viable BM-MSCs after 24 h between the GEM (10 μM) vs. GEM + FLU (10 μM + 10 μM), GEM (10 μM) vs. GEM + FLU (10 μM + 2 mM), and GEM + FLU (10 μM + 10 μM) vs. GEM + FLU (10 μM + 2 mM) groups was *p* > 0.5, *p* < 0.001, and *p* < 0.001, respectively. The details of the statistical analysis are presented in Table S1 of the Supplementary Materials.

Based on the presented findings, it seems that BM-MSCs after 24 h can be applied as a potential GEM + FLU carrier. In fact, the Western blot study did not show CASP3 activation in the BM-MSC line after treatment with GEM (10 μM) and GEM + FLU (10 μM + 10 μM); therefore, the above cell line was selected for further experiments.

### 2.5. The Influence of Cytotoxic Activity and the Ability to Activate Apoptosis and Necrosis After Treatment with BM-MSC-Conditioned Media in PCCs

**Figure 8:**
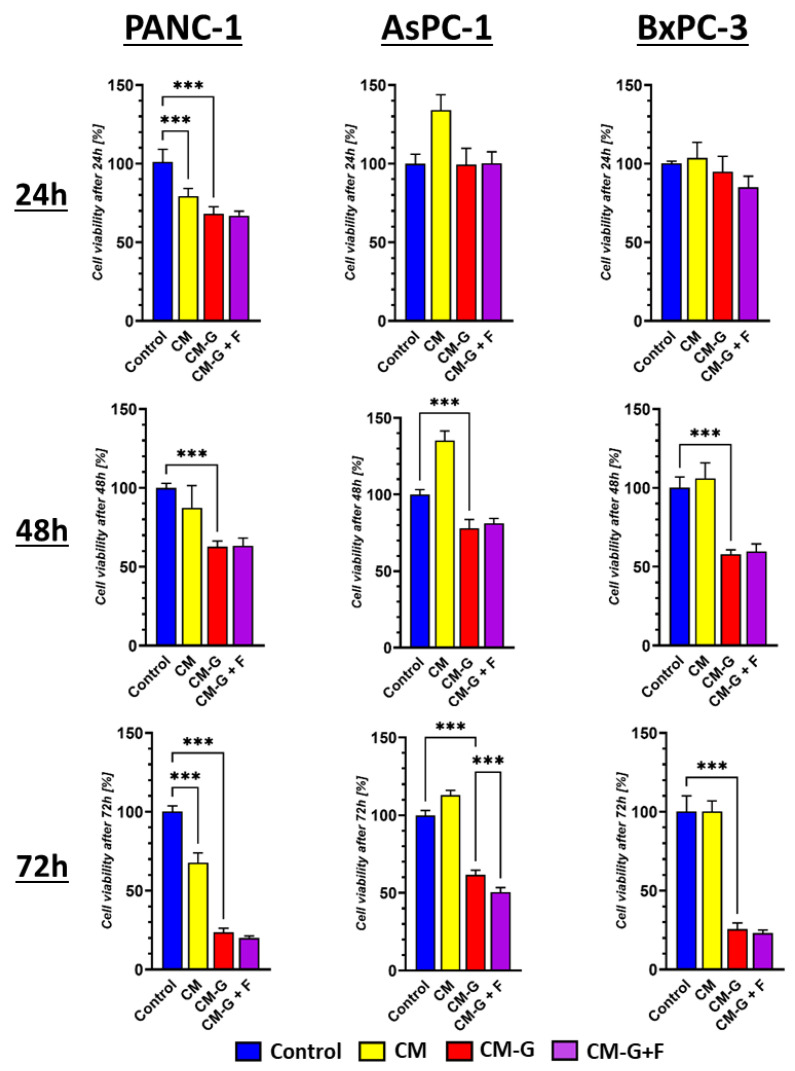
An MTS assay was performed on PCCs cultured with CM (yellow), CM-GEM (red), and CM-GEM + FLU (purple) for 24, 48, and 72 h. Conditioned media were diluted 1:1 with the fresh medium. The effect was measured by an MTS assay. Statistical analysis was performed using Welch’s ANOVA with Brown–Forsythe correction, followed by Dunnett’s T3 multiple comparisons test comparing control vs. CM, control vs. CM-GEM, and CM-GEM vs. CM-GEM + FLU (* *p* ≤ 0.05, ** *p* ≤ 0.01, *** *p* ≤ 0.001). The data are shown as mean ± SD of the triplicate experiments.

The tested conditioned media from GEM- and FLU-treated and untreated BM-MSCs (CM, CM-GEM, and CM-GEM + FLU) were diluted 1:1 with the fresh medium before the treatment with PCCs. A decrease in the viability of pancreatic cancer cell lines was observed after treatment with CM-GEM and CM-GEM + FLU, as illustrated in Figure 8.

The percentage of viable cells after 72 h was 23.5 (±2.6)% for CM-GEM; 20 (±1.22)% for CM-GEM + FLU for PANC-1; 61.55 (±3.04)%; 50.4 (±2.96)% for AsPC-123 and 25.66 (±3.9)%; and 23.00 (±2.06)% for BxPC-3, respectively. FLU addition did not significantly affect the survival of PANC-1 and BxPC-3. In contrast, CM treatment promoted the growth of AsPC-1 tumor cells. We observed a significant increase in the AsPC-1 cell population at all observed time points following CM treatment (*p* < 0.001). At 24, 48, and 72 h, the number of cells increased by 34.0% (±9.78), 35.11% (±6.4), and 13.22% (±3.0), respectively.

**Figure 9:**
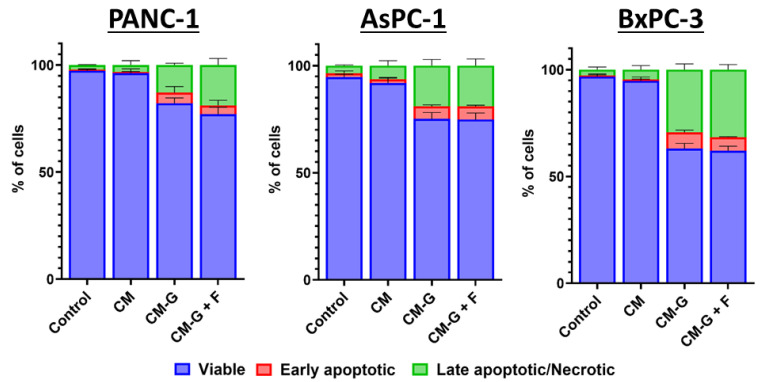
PCC apoptosis and necrosis after 72 h CM, CM-GEM, and CM-GEM + FLU treatment (Annexin V and 7-AAD, flow cytometry). The graphs show the percentage of viable (blue), early apoptotic (red), and late apoptotic/necrotic (green) cells after treatment with CM or CM with drugs. Data were analyzed using one-way ANOVA, followed by Sidak’s multiple comparisons test comparing CM vs. CM-GEM/CM-GEM + FLU and CM-GEM vs. CM-GEM + FLU. The data are shown as mean ± SD of the triplicate, independent experiments.

A significant increase in the percentage of apoptotic and necrotic cells was observed after CM-GEM and CM-GEM + FLU treatment of PCCs (see Figure 9).

For CM-GEM, the average percentage of live cells after 72 h was 84.27% for PANC-1; 78.64% for AsPC-1; and 65.05% for BxPC-3, while, for CM-GEM + FLU, it was 80.83%, 77.63%, and 64.16%, respectively. The statistical significance calculated for live cells between the control group vs. CM-GEM and the control group vs. CM-GEM + FLU was *p* < 0.001 after 72 h for all PCCs. On the contrary, higher *p* values were recorded between CM-GEM and CM-GEM + FLU with *p* > 0.6 for PANC-1, *p* > 1 for AsPC-1, and *p* > 0.8 for BxPC-3. No significant effect of the GEM + FLU combination and conditioned medium from BM-MSCs or an effect of CM on the percentage of viable PCCs was observed (see statistical data in Table S2 of the Supplementary Materials).

### 2.6. Co-Culture of GEM- and FLU-Treated BM-MSCs with PCCs

**Figure 10:**
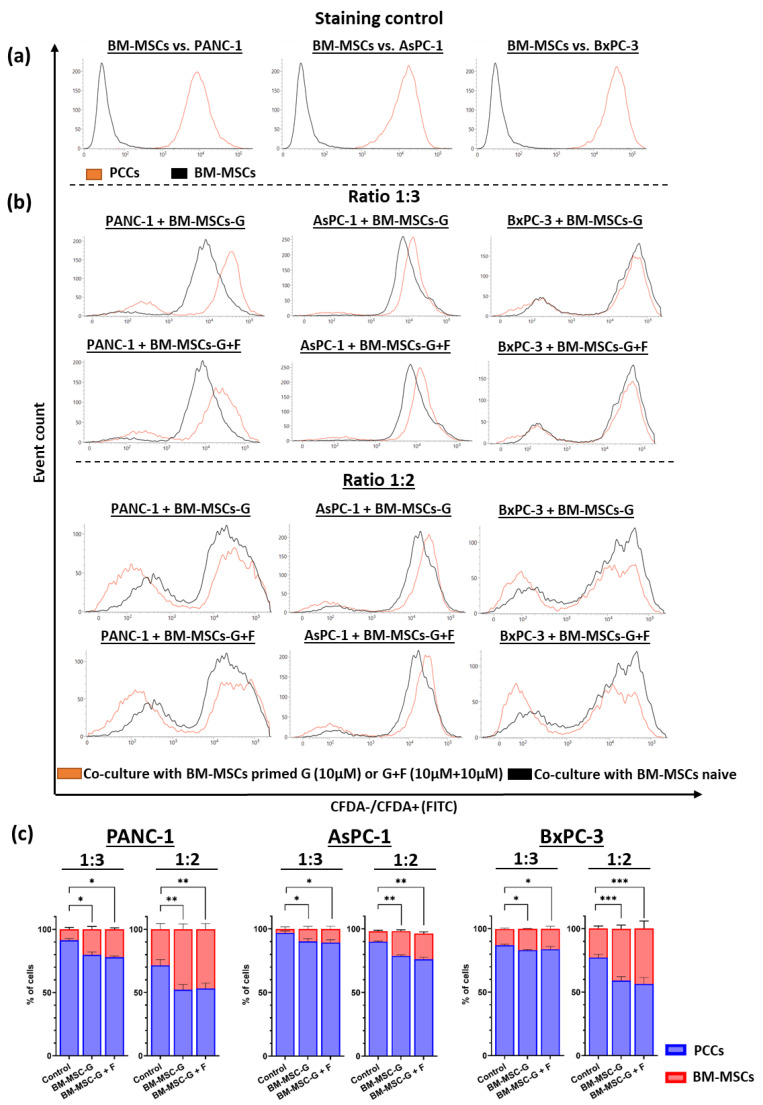
PCC and BM-MSC co-culture after GEM (10 μM) and GEM + FLU (10 μM + 10 μM) treatment of BM-MSCs. This study was performed after staining PCCs with CFDA (flow cytometry): (**a**) control of PCCs (orange line) and BM-MSCs (black line) staining; (**b**) comparison of untreated co-culture (black line) and tested co-culture (BM-MSCs-GEM or BM-MSCs-GEM + FLU) (orange line) in a ratio of 1:3 or 1:2 (BM-MSCs:PCCs); (**c**) percentage of cells of the respective PCC and BM-MSC lines depending on the drug treatment conditions. Statistical significance was performed using the Holm–Sidak post hoc test comparing control vs. GEM/GEM + FLU and GEM vs. GEM + FLU (* *p* ≤ 0.05, ** *p* ≤ 0.01, *** *p* ≤ 0.001) for the percentage of PCCs between the groups. The data are shown as mean ± SD of the triplicate experiments.

Quantitative analysis of PANC-1, AsPC-1, and BxPC-3 cells stained with CFDA was performed after 24 h co-culture with untreated and GEM (10 μM) or GEM + FLU (10 μM + 10 μM)-treated BM-MSCs in a ratio of 1:3 and 1:2 (BM-MSCs:PCCs), as presented in Figure 10. BM-MSCs treated with GEM, both alone and in combination with FLU, induce a cytostatic effect on the tested PCCs. In particular, the cytostatic effect was visible after adding treated BM-MSCs in a ratio of 1:2, as shown in Figure 10b. On the other hand, the untreated BM-MSCs (used in ratios of 1:2 and 1:3) stimulated the growth of tumor cells. The addition of FLU did not enhance the effect of GEM, and no significant differences were found between the GEM (10 μM) and GEM + FLU (10 μM + 10 μM) samples (see Figure 10b).

### 2.7. Western Blot Technique

**Figure 11:**
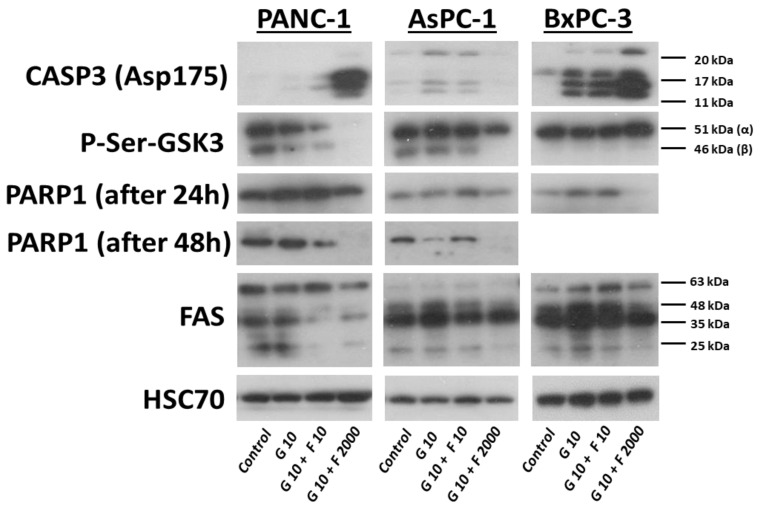
The expression of CASP3, P-Ser-GSK3α and β, FAS, and HSC70 (control) after GEM (10 μM), GEM + FLU (10 μM + 10 μM), and GEM + FLU (10 μM + 2 mM) treatment for 24 h. The expression of PARP1 is presented after 24 h and 48 h. This study was performed using the Western blot for PCCs.

The expression of caspase-3, PARP1, p-Ser-GSK3β, and FAS proteins was examined in the selected PCCs after treatment with GEM (10 μM), GEM + FLU (10 μM + 10 μM), GEM + FLU (10 μM + 2 mM), and media conditioned with CM, CM-GEM, and CM-GEM + FLU after 1:1 dilution with fresh medium. Caspase-3 expression significantly increased in the PANC-1 line after treatment with GEM + FLU (10 µM + 2 mM), which indicates an intensive activation of the apoptotic process. No changes in PARP1 were observed after 24 h compared to the control, however, after 48 h, it was undetectable in the case of the highest dose of drugs (10 µM + 2 mM). The expression of p-Ser-GSK3β decreased, especially after using the combination of GEM + FLU (10 µM + 2 mM), which indicates the activation of GSK3β. In turn, the reduced expression of FAS in the combination of GEM + FLU (10 µM + 2 mM) may suggest changes in the signaling that depend on cell death receptors and blocking the FAS-based death pathway, as shown in Figure 11.

**Figure 12:**
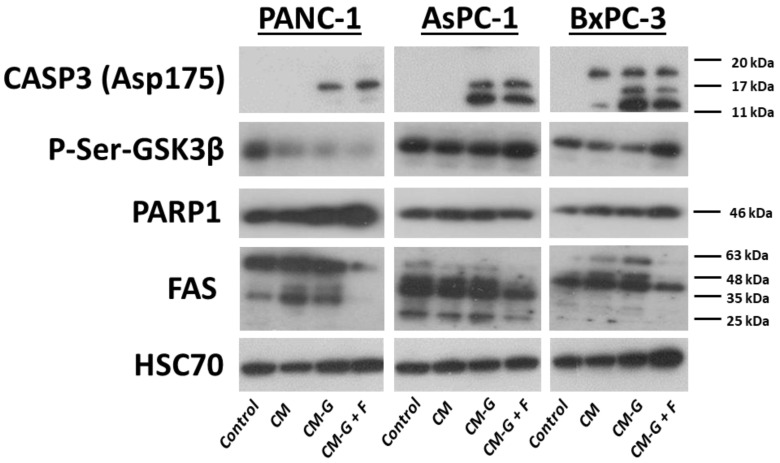
The expression of CASP3, P-Ser-GSK3α and β, PARP1, FAS, and HSC70 (control) after CM, CM-GEM, and CM-GEM + FLU treatment for 72 h was recorded using the Western blot for PCCs.

Despite lower doses of used drugs, caspase-3 was still clearly detectable after the treatment of PANC-1 cells with CM-GEM and CM-GEM + FLU. The increased expression of PARP1 was also observed compared to the control and CM; however, p-Ser-GSK3β was undetectable in CM-GEM and CM-GEM + FLU treatments. Similarly to the treatment with single drugs, FAS expression also decreased after CM-GEM + FLU treatment, as presented in Figure 12.

Caspase-3 was present after GEM (10 μM) and GEM + FLU (10 μM + 10 μM) treatment in AsPC-1 cells, whereas it was not visible after the application of the GEM + FLU (10 μM + 2 mM) combination. After GEM + FLU (10 μM + 2 mM) treatment, p-Ser-GSK3β expression was undetectable, while p-Ser-GSK3α was at a lower level compared to that of the control, as well as the other drug combinations. No changes in PARP1 were observed after 24 h compared to the control, however, after 48 h, its amount decreased to an undetectable level for the GEM + FLU (10 μM + 2 mM) treatment. The administration of a higher dose of drugs resulted in the reduction in FAS expression after the treatment (see Figure 11). After the addition of a conditioned medium, caspase-3 was clearly visible (CM-GEM and CM-GEM + FLU), indicating the presence of apoptosis to a greater extent compared to the usage of the singular drugs, but p-Ser-GSK3β and PARP1 showed no visible changes in expression. In contrast, FAS was at undetectable levels after CM-GEM + FLU treatment (see Figure 12). Noticeably, applying the GEM + FLU (10 μM + 2 mM) combination led to strong activation of caspase-3 for the BxPC-3 line, while the expression of p-Ser-GSK3α remained stable under all conditions. In fact, PARP1 expression was not visible after 24 h of using the GEM + FLU (10 μM + 2 mM) combination, indicating strong activation of the apoptosis process. FAS showed a higher expression with GEM and GEM + FLU (10 μM + 10 μM), while it decreased at the highest dose of GEM + FLU (10 μM + 2 mM). CM-GEM and CM-GEM + FLU treatments also increased caspase-3 expression, despite the lower dose of used drugs. The level of p-Ser-GSK3β increased after CM-GEM + FLU treatment (indicating inhibition of GSK3β), which might suggest a completely different mechanism of regulation compared to direct drug treatment. PARP1 expression increased, which may indicate enhanced DNA repair processes. Similarly to using singular drugs, FAS protein expression grew after CM-GEM treatment. At the same time, it disappeared for CM-GEM + FLU, pointing out the specific changes in the signaling pathway dependent on cell death receptors in this cell line after additional FLU treatment.

## 3. Discussion

Not only the application of FLU as a GEM-supporting agent in the anticancer therapy of pancreatic cancer but also the involvement of BM-MSCs as potential drug carriers is presented here. As a matter of fact, the sensitizing effect of aspirin on gemcitabine-resistant cells, such as AsPC-1 and PANC-1, has been discussed meticulously [51,52,57]. To the best of our knowledge, the efficacy of FLU (as another NSAID drug that enhances the cytotoxicity of gemcitabine) has not been presented yet. Our results show that the combination of GEM + FLU induces a higher cytotoxic effect on PCCs compared to GEM alone. Cell cycle analysis proved the existence of the synergistic GEM + FLU effect. The arrest of the cell cycle in the G1 phase (particularly visible in the PANC-1 and AsPC-1 lines) indicates the possibility of inducing antiproliferative effects by FLU, which may be beneficial in combined therapy. The presence of BxPC-3 cells in the sub-G1 phase suggests that the combination of GEM and FLU may induce apoptosis in these cells through DNA fragmentation mechanisms. The examination of annexin-V and 7-AAD confirmed significantly higher levels of apoptotic and necrotic cells in all PCCs tested after applying the GEM + FLU combination, especially at higher FLU concentrations (2 mM). The obtained findings suggest the higher efficacy of the combination therapy, compared to GEM alone.

The analysis of BM-MSC survivability and the examination of the caspase-3 assay revealed lower sensitivity of these cells to GEM (especially after 24 h), which promotes the usage of BM-MSCs as potential drug carriers. Nevertheless, at higher concentrations or after more prolonged exposure (48 h), a noticeable depletion in BM-MSC survivability was recorded (especially for the GEM + FLU combination). The observed tendency is in line with previous findings, indicating limited cytotoxicity of FLU towards normal cells, as well as its ability to potentiate the action of anticancer drugs in cancer cells simultaneously [58].

MSCs are known to actively secrete EVs, including exosomes and larger apoptotic bodies, which play a crucial role in intercellular communication. These microvesicles are formed in the process of intracellular membrane budding, afterwards being released into the extracellular environment, serving as bioactive molecule transporters (e.g., mRNA, miRNA, lncRNA, proteins, lipids, and metabolites). In consequence, MSC microvesicles can modulate biological processes in target cells, affecting the mechanisms of regeneration, inflammation, and immunomodulation, as well as apoptosis and proliferation of cancer cells. Due to the natural biocompatible properties and tropism to damaged/cancerous tissues, MSCs can serve as potential drug delivery agents, therapeutic genes, or inhibitors of specific molecular pathways. Considering the EVs’ properties, we decided to investigate the impact of the medium conditioned from BM-MSCs treated with GEM and FLU on selected PCCs. The undertaken studies aimed to find out whether the EVs contained in the conditioned medium, as well as other factors secreted by BM-MSCs in response to drug action, can mediate their antitumor effects. Based on the experimental findings, it is suggested that CM-GEM can affect the viability of pancreatic cancer cells and induce apoptotic and necrotic mechanisms even at relatively low drug concentrations. Interestingly, the addition of FLU to CM-GEM did not significantly enhance the toxic effect; however, the ability of gemcitabine metabolites to exhibit antitumor activity is still controversial. It is believed that gemcitabine diphosphate (dFdCDP) and triphosphate (dFdCTP), formed intracellularly, are responsible for the antitumor activity of gemcitabine. As a result of deamination, the majority of gemcitabine is converted into inactive 2′,2′-difluoro-2′-deoxyuridine (dFdU), which may limit GEM activity. In fact, dFdU can also be phosphorylated in the cell to form the active forms of monophosphate (dFdUMP), diphosphate (dFdUDP), and triphosphate (dFdUTP). Furthermore, the contribution of dFdU nucleotides to antitumor activity has also been suggested [59,60].

A proliferation analysis of AsPC-1 lines after CM treatment showed increased cell viability, which confirms the growth-promoting properties of BM-MSCs. It is known that BM-MSCs are able to secrete cytokines and growth factors that can support the survival and proliferation of cancer cells [61,62]; however, the presence of GEM limits these properties, which allows for the potential use of BM-MSCs as transporters of anticancer drugs. Apoptosis and necrosis examination after applying CM-GEM or CM-GEM + FLU revealed significant growth in the percentage of apoptotic and necrotic cells in PANC-1, AsPC-1, and BxPC-3. Roughly speaking, the conditioned medium obtained by the action of GEM on BM-MSCs may induce cytotoxic effects directly in pancreatic cancer cells. It should be emphasized that the addition of FLU did not significantly enhance the effect of apoptosis, which suggests the dominant impact of GEM in inducing cell death phenomenon.

Additionally, the influence of BM-MSC co-cultures on pancreatic cancer cells was investigated. It was confirmed that treated BM-MSCs can inhibit the growth of the cell lines, especially in a ratio of 1:2 (BM-MSCs:PCCs), which is in line with the hypothesis that BM-MSCs treated with anticancer drugs can serve as potential therapeutic vectors supporting cytotoxicity [63]. On the other hand, BM-MSCs not treated with drugs showed properties promoting the growth of cancer cells, especially the AsPC-3 line, which confirms their dualistic properties as well [64].

Comparing the treatment response of the selected PCCs to the combination of GEM + FLU, the variations in the activation of apoptosis pathways were revealed depending on the presence and dose of FLU; therefore, the observed phenomena may be related to the different sensitivity of these cells to therapy. Practically, an increased expression of proteins responsible for initiating apoptosis pathways was recorded after using the GEM + FLU combination.

Apoptosis is most intense at the GEM + FLU dose (10 μM + 2 mM) for the PANC-1 line, which is confirmed by the strong activation of caspase-3 (one of the key effectors of apoptosis) and the degradation of PARP1. In fact, FAS expression was not demonstrated after the administration of the drug combination; however, the AsPC-1 and BxPC-3 lines react differently to the treatment with the compounds. In the case of AsPC-1, the apoptotic response is weaker, and some markers (such as caspase-3 and FAS) remain less active, which may indicate a greater resistance of the cell line. A visible activation of apoptosis through the strong expression of caspase-3 and the degradation of PARP1 after 24 h of FLU addition is observed, while no expression of p-Ser-GSK3β was demonstrated (apoptotic pathways may be differentially regulated, independently of GSK3β). Such variations in response to treatment may indicate specific mechanisms of resistance, affecting the therapy efficacy for different types of pancreatic cancers.

In turn, the analysis of the PCCs’ response to the action of the conditioned medium (CM), the medium containing gemcitabine metabolites (CM-GEM), and gemcitabine and flurbiprofen metabolites (CM-GEM + FLU) reveals further differences in the activation of apoptosis and signaling pathway involvement. The cytotoxic effect of CM-GEM and CM-GEM + FLU is most visible in the BxPC-3 line. A decrease in the survival of BxPC-3 cells and the activation of caspase-3 were also recorded after the application of CM alone; moreover, similar findings were recorded when the drugs were used alone at higher doses. In PANC-1, apoptosis is weaker after the usage of CM, CM-GEM, and CM-GEM + FLU, whereas, a greater involvement of caspase-3 can be observed in the AsPC-1 line, compared to the drugs when used alone. In contrast, the FAS pathway is strongly suppressed in the presence of flurbiprofen, which may suggest an advanced stage of apoptosis (FAS is degraded) or a reduced activity of the FAS-dependent pathway. On the other hand, the extrinsic death-receptor-dependent apoptosis pathway does not play a dominant role in the response of these cells to treatment [65]. Appealing findings were received in the expression of p-Ser-GSK3, which disappears in PANC-1 with an increasing FLU concentration (compared to drugs when used alone), suggesting the activation of GSK3β, which might result in the related effect on cell survival. In the BxPC-3 cell line, p-Ser-GSK3β expression remained stable when the drugs were administered alone. In contrast, an increase in phosphorylation and blocking of GSK3β function (which may accompany apoptosis) was observed after CM-GEM/CM-GEM + FLU treatment. It should be highlighted that GSK-3β is strongly expressed in many cancers, being involved in the regulation of proliferation, apoptosis, and chemoresistance. On the other hand, different roles of GSK-3β as an anticancer and tumor-development promoting agent have been reported so far; therefore, applying drugs targeting GSK-3β in cancer treatment seems controversial. In spite of the fact that the role of GSK-3β in cancer cells as a pro-oncogenic or suppressor protein is debatable, it has been agreed that GSK3-β is a pro-tumor protein and a good candidate for the targeted treatment of pancreatic cancers [66].

In this study, we found that GEM + FLU mediates apoptosis and the inhibition of proliferation by affecting key proteins such as caspase-3, PARP1, GSK3β, and FAS. However, many proteins involved in these pathways are not fully understood. One possibility to combat this is to use artificial intelligence. AI models, such as AlphaFold, are able to predict the structure and functional domains of key proteins and their interactions with membrane receptors in cancer cells. It is planned to use AI models to predict protein–ligand or protein–protein geometries, which might provide profound insight into the GEM and FLU interactions. The proven efficacy of AI models in similar studies gives the opportunity to enrich our further studies with information on the potential mechanisms of GEM-FLU interaction. Consequently, it may be helpful in planning and developing a personalized therapeutic strategy [67,68]. The obtained results suggest that the combination of GEM with FLU may represent a promising therapeutic strategy not only for pancreatic cancer, but also for other types of cancer. To the best of our knowledge, the efficacy of FLU, as another NSAID that enhances the cytotoxicity of gemcitabine, was reported only once and showed the strongest anticancer effect compared to other NSAIDs [56]. Nevertheless, our study has several limitations. While in vitro experiments are a valuable tool for testing new drug combinations, they have limited ability to accurately predict drug efficacy in patients, cannot fully replicate the complex tumor microenvironment, and are susceptible to cellular changes during culturing. Additionally, the heterogeneity of BM-MSCs may also present a challenge. This highlights the need for new strategies to purify MSCs for future standardized production and reliable clinical application. Future in vivo studies using appropriate animal models will be essential to validate the in vitro findings, optimize dosing strategies, reduce potential side effects, and identify any unforeseen biological responses to the investigated drug combination.

## 4. Materials and Methods

### 4.1. Reagents

Gemcitabine (GEM) and flurbiprofen (FLU) were purchased from Angene Chemical (Nanjing, Jiangsu, China) dissolved in DMSO (200 mg/mL stock), and stored at a temperature of −20 °C. Dilutions (1 mM or 10 mM in PBS) were prepared just before application.

### 4.2. Cell Lines

Human pancreatic cancer cell lines (PCCs), PANC-1 and AsPC-1, were purchased from ATCC, and BxPC-3 was obtained from the Cell Bank of the Maria Sklodowska-Curie NRIO (courtesy of Prof. Ryszard Smolarczyk, NRIO, Poland). PANC-1 cells were cultured in DMEM containing 10% fetal bovine serum (FBS) (Biowest SAS, Nuaille, France) and 1% penicillin/streptomycin (Gibco, Thermo Fisher Scientific Inc., Waltham, MA, USA). AsPC-1 and BxPC-3 cells were cultured in RPMI-1640 (Biowest SAS, Nuaille, France) supplemented with 10% FBS, 2% GlutaMax (Gibco, Thermo Fisher Scientific Inc., Waltham, MA, USA), and 1% penicillin/streptomycin (Gibco, Thermo Fisher Scientific Inc., Waltham, MA, USA). The cultures were grown in a humidified atmosphere at 37 °C with 5% CO_2_ and were routinely tested for mycoplasma contamination. The culture medium was replaced every 2–3 days, and cell passage was performed when cell confluence reached 70–80%.

### 4.3. BM-MSC Isolation and Phenotyping

Bone marrow samples collected from healthy donors (Maria Sklodowska-Curie NRIO, Poland) were diluted with Minimal Eagle’s Medium (MEM) supplemented with 10% human platelet lysate (hPLT), 1% penicillin/streptomycin (Gibco; Thermo Fisher Scientific Inc.), 1% non-essential amino acids (Gibco; Thermo Fisher Scientific Inc.), and 2 U/mL of heparin (Polfa, Warsawa, Poland). These samples were then transferred to a humidified incubator set at 37 °C with 5% CO_2_. After 24 h, the cultures were washed with PBS to remove non-adherent cells. Sub-confluent cultures were passaged at a 1:3 split ratio, and cells from passages 2 to 5 were used for subsequent experiments.

To confirm the isolation of bone-marrow-derived mesenchymal stem cells (BM-MSCs), the cells were screened using the Human MSC Analysis Kit (BD Biosciences, San Jose, CA, USA) to detect MSC-associated surface markers (CD73, CD90, and CD105), and to ensure the absence of blood-cell-lineage-specific markers (CD11b, CD19, CD34, CD45, and HLA-DR). For this purpose, the cultured cells were collected, washed with PBS, and incubated with appropriate antibodies for 20 min at a room temperature. Following incubation, the cells were washed with Cell Wash Buffer (BD Biosciences) and suspended. Analysis was conducted using a flow cytometer (BD FACSLyric, San Jose, CA, USA). Gating parameters to distinguish positive and negative populations were established based on the signals from the isotype IgG control probes.

### 4.4. Proliferation Assays

PANC-1, AsPC-1, BxPC-3, and BM-MSC lines were seeded into 96-well plates in appropriate media at a density of 4 × 10^3^ cells per well. After 24 h of incubation, the cells were treated with GEM at concentrations ranging from 0.01 μM to 100 μM; GEM + FLU at equal concentrations ranging from 0.01 μM to 100 μM; and GEM + FLU at different concentrations using a much higher dose of FLU (1 μM + 1 mM; 10 μM + 1 mM; 1 μM + 2 mM; and 10 μM + 2 mM). BM-MSCs were treated with GEM at concentrations ranging from 0.01 μM to 100 μM; FLU at concentrations ranging from 0.01 μM to 100 μM; and GEM + FLU at equal concentrations ranging from 0.01 μM to 100 μM. The control consisted of untreated cells. The cells were incubated for 24 h and 48 h at 37 °C and 5% CO_2_. Then, the MTS reagent (CellTiter 96^®^ AQueous Non-Radioactive Cell Proliferation Assay, Promega Corporation, Madison, WI, USA) was added to the wells and incubated for 1.5 h. Absorbance was then measured spectrophotometrically at 490 nm using a microplate reader. The percentage of viable cells was calculated according to the following formula:

(1) $$\frac{Absorbance\;of\;samples\;\left[nm\right]-Absorbance\;of\;blank\;[nm]}{Absorbance\;of\;control\;\left[nm\right]-Absorbance\;of\;blank\;[nm]}\times 100$$

### 4.5. Annexin V and 7-AAD Assay

PANC-1, AsPC-1, BxPC-3, and BM-MSC lines were seeded in 6-well plates at 2 × 10^5^ cells per well. After 24 h of incubation, the cells were treated with GEM 10 μM; GEM + FLU at an equal concentration (10 μM + 10 μM); and GEM + FLU at different concentrations (10 μM + 2 mM). The cells were incubated for 2 h and 48 h at 37 °C and 5% CO_2_. Next, the cells were stained with annexin V and 7-AAD (FITC Annexin V Apoptosis Detection Kit with 7-AAD(BioLegend Inc., San Diego, CA, USA). Apoptotic and necrotic cells were detected using flow cytometry (BD FACSLyric, San Jose, CA, USA).

### 4.6. Cell Cycle

PANC-1, AsPC-1, and BxPC-3 cell lines were seeded in 6-well plates at 2 × 10^5^ cells per well. After 24 h of incubation, the cells were treated with GEM 10 μM; GEM + FLU at an equal concentration (10 μM + 10 μM); and GEM + FLU at different concentrations (10 μM + 2 mM). The untreated cells served as a control. The cells were incubated for 24 h at 37 °C and 5% CO_2_ and were trypsinized and fixed with 70% ethanol overnight at 4 °C. Then, the alcohol was removed, and 100 μg/mL RNAse (EURx Sp. z o.o., Gdańsk, Poland) and 100 μg/mL propidium iodide (Merck Life Science KGaA, Darmstadt, Germany) were added. Then, the cells were incubated in the dark (20 min., RT) followed by flow cytometry (BD FACSLyric)

### 4.7. BM-MSC Pre-Treatment with GEM and FLU and Conditioned Media Collection

BM-MSC priming was performed according to the protocol of Bonomi et al. [12,69].

GEM and FLU (1 mM) solutions were used in two combinations: GEM 10 μM and GEM + FLU (10 μM + 10 μM). Each solution was added to the culture medium without hPLT to obtain appropriate concentrations. The concentrations were determined based on the MTS assay of BM-MSCs and PCCs PANC-1, AsPC-1, and BxPC-3 cells. The drugs were administered to sub-confluent BM-MSCs. After 24 h of incubation, the culture medium was removed, and the cells were washed twice with PBS to remove free drugs. Cells were trypsinized, and the number and viability were assessed (DAPI staining, BD FACSLyric flow cytometer). Then, 1 × 10^6^ cells were suspended in a medium without hPLT and transferred to a new 75 cm^2^ culture flask. After 24 h, the conditioned media were collected and filtered through a 0.22 µm filter. The following conditioned media were obtained: medium from untreated BM-MSCs (CM), medium from BM-MSCs treated with GEM (CM-GEM), medium from BM-MSCs treated with GEM, and FLU (CM-GEM + FLU). The collected conditioned media were stored at −70 °C. BM-MSCs were trypsinized and washed twice with PBS, which resulted in the preparation of drug-free BM-MSCs (BM-MSCs), BM-MSCs with GEM (BM-MSCs-GEM), and BM-MSCs with GEM and FLU (BM-MSCs-GEM + FLU), which were used to prepare the co-culture with PCCs.

### 4.8. Proliferation Assay of BM-MSC-CM Priming with GEM or GEM + FLU

PANC-1, AsPC-1, and BxPC-3 cell lines were seeded in 96-well plates at 2 × 10^3^ cells per well. After 24 h of incubation, the cells were treated with a conditioned medium from BM-MSCs:CM, CM-GEM, or CM-GEM + FLU. The conditioned media were diluted 1:1 with the medium appropriate for the given cell line. After 24 h, 48 h, and 72 h of incubation, the MTS reagent was added to the wells and incubated for 1.5 h. Then, it was measured spectrophotometrically at 490 nm using a microplate reader. The percentage of viable cells was calculated according to Formula (1).

### 4.9. Annexin V and 7-AAD Assay of BM-MSC-CM Priming with GEM or GEM + FLU

PANC-1, AsPC-1, and BxPC-3 cells were incubated for 72 h in a conditioned medium from BM-MSCs:CM, CM-GEM, or CM-GEM + FLU. The conditioned media were diluted 1:1 with an appropriate medium for a given cell line. Then, the cells were trypsinized and stained with annexin V and 7-AAD (FITC Annexin V Apoptosis Detection Kit with 7-AAD(BioLegend Inc., San Diego, CA, USA). Apoptotic and necrotic cells were detected using flow cytometry (BD FACSLyric)

### 4.10. CFDA Staining

PANC-1, AsPC-1, and BxPC-3 cells were collected, washed once with PBS, centrifuged, and gently resuspended in PBS containing CFDA (Vybrant™ CFDA SE Cell Tracer Kit, Invitrogen, Thermo Fisher Scientific Inc.; Paisley, Scotland) at a concentration of 5 μM/mL. The cells were incubated for 15 min at 37 °C, then centrifuged and resuspended in a fresh medium. The cells were counted and seeded onto chamber slides at a density of 20,000 cells per well. After 24 h, the cells were divided into groups and treated with the following: GEM 10 μM; GEM + FLU and in equal concentrations (10 μM + 10 μM); and GEM + FLU at different concentrations (10 μM + 2 mM). After 24 h, the cells were washed and fixed with cold 70% ethanol. For cell nucleus staining, DAPI counterstain was used. Photographs were taken using a fluorescence microscope (Carl Zeiss Microscopy GmbH, Jena, Germany).

### 4.11. BM-MSCs Co-Culture

PANC-1, AsPC-1, and BxPC-3 cells stained with CFDA were seeded in 6-well plates at 1 × 10^5^ cells per well. The next day, native BM-MSCs (control), BM-MSCs-GEM, or BM-MSCs-GEM + FLU cells were added at 1:2 and 1:3. After 24 h, the co-cultures were harvested, and the cells were detected using flow cytometry (BD FACSLyric).

### 4.12. Western Blotting

PANC-1, AsPC-1, and BxPC-3 cell lines were treated with GEM 10 μM; GEM + FLU at an equal concentration (10 μM + 10 μM); or GEM + FLU at different concentrations (10 μM + 2 mM) for 24 h and with CM, CM-GEM, CM-GEM + FLU diluted 1:1 with the medium intended for a given cell line for 72 h, and then lysates were prepared. Whole-cell lysates were prepared using an IP buffer supplemented with protease and phosphatase inhibitors, as described previously [70]. Aliquots of lysates (15–40 µg) were separated by SDS-PAGE on 8% or 13% gels and electrotransferred to PVDF membranes. Prior to incubation with primary antibodies, the membranes were incubated for 1 h at room temperature in a blocking solution (5% skimmed milk in PBS containing 0.1% Tween-20). Anti-cleaved caspase-3 (Asp175) (5A1E) rabbit mAb, anti-P-Ser-GSK3B rabbit mAb, and anti-Fas rabbit mAb were purchased from Cell Signaling Technology (Danvers, MA, USA). Anti-PARP1 and loading control anti-HSC70 (B-6) antibodies were purchased from Santa Cruz Biotechnology (Dallas, TX, USA). All primary antibody incubations were performed overnight at 4 °C in a blocking solution. HRP-conjugated secondary antibodies (anti-mouse and anti-rabbit) were detected by chemiluminescence (SuperSignal West Pico or SuperSignal West Femto Chemiluminescent Substrate, Thermo Fisher Scientific Inc., Waltham, MA, USA) to generate a visible signal. Chemiluminescent detection was performed using the OPTIMAX automated X-Ray Film Processor. For molecular weight determination, the Thermo Fisher PageRuler Prestained Protein Ladder (10 to 180 kDa) (Thermo Fisher Scientific Inc., Waltham, MA, USA was used, which does not contain any epitopes or enzymes that would allow light emission upon chemiluminescent substrate exposure. Therefore, the marker is not visible on the final chemiluminescent images. The marker was present on the membranes and used to determine the molecular weights of the detected proteins.

### 4.13. Statistical Analysis

All experiments were repeated at least three times. Data are presented as mean ± standard deviation (SD). Statistical analyses were performed using GraphPad Prism version 10.0.0 for Windows (GraphPad Software, Boston, MA, USA, www.graphpad.com, accessed on 14 May 2024). One-way analysis of variance (ANOVA) was used for the comparison of multiple data groups, where tests with * *p* ≤ 0.05, ** *p* ≤ 0.01, and *** *p* ≤ 0.001 were regarded as statistically relevant. If data did not meet the assumption of variance homogeneity (checked using the Brown–Forsythe test), the Welch’s counterpart of the ANOVA was used with Dunnett’s T3 test implemented for post hoc comparisons. Otherwise, the classical one-way ANOVA was engaged, with the Sidak’s test or Holm–Sidak’s tests being used for comparisons between selected groups (e.g., single drug vs. combination of drugs).

## 5. Conclusions

Based on the experimental and statistical data, the following conclusions can be drawn:

- An interesting application of flurbiprofen as an adjuvant of gemcitabine in vitro inhibiting the growth of pancreatic cancer cells is presented;
- The combination of GEM + FLU showed significantly higher cytotoxicity towards PANC-1, AsPC-1, and BxPC-3 cells compared to gemcitabine alone;
- FLU enhanced the antiproliferative effect of GEM, inducing apoptosis and inhibiting the cell cycle;
- Western blot confirmed an increased expression of caspase-3 and a decrease in p-Ser-GSK3β and PARP1;
- The conditioned medium from BM-MSCs with drugs led to apoptosis, irrespective of the lower drug dose usage; moreover, possible interactions of BM-MSCs with cancer cells require further study;
- The combination of flurbiprofen and gemcitabine might be regarded as a potentially effective strategy in the therapy of pancreatic cancer, especially in the context of proliferation inhibition and the induction of cancer cell death.

## Figures and Tables

**Table 1:** PCC and BM-MSC IC_50_ values for GEM and GEM + FLU treatment in equal concentrations after 48 h.

Compound	IC_50_ [μM] ± SD			
	PANC-1	AsPC-1	BxPC-3	BM-MSCs
GEM	228.2 ± 16.3	194.6 ± 11.2	98.6 ± 8.8	240 ± 19.8
GEM + FLU	109.3 ± 9.6	104.3 ± 7.3	1.9 ± 0.5	179.1 ± 13.5

## Data Availability

The data presented in this study are available upon request from the corresponding author.

## Supplementary Materials

The following supporting information can be downloaded at: https://www.mdpi.com/article/10.3390/ijms26136212/s1

## Abbreviations

The following abbreviations are used in this manuscript:

GEM	Gemcitabine
FLU	Flurbiprofen
BM-MSCs	Bone-marrow-derived mesenchymal stem cells
NSAIDs	Nonsteroidal anti-inflammatory drugs
CM	Conditioned medium
EVs	Extracellular vehicles
PDAC	Pancreatic ductal adenocarcinoma
NTs	Nucleoside transporters
