# Supplementary material for: Gemcitabine and Flurbiprofen Enhance Cytotoxic Effects on Cancer Cell Lines Mediated by Mesenchymal Stem Cells
Source: Int J Mol Sci. 2025 Jun 27;26(13):6212. doi: 10.3390/ijms26136212 (PMC12250333; doi:10.3390/ijms26136212)
Supplement: Supplementary file 1 [file ijms-26-06212-s001.zip › ijms-3637923-supplementary.pdf]

Supplementary materials

Gemcitabine and Flurbiprofen Enhance Cytotoxic Effects on Cancer Cell Lines Mediated by Mesenchymal Stem Cells

Agata Kawulok [1,2] *, Paulina Borzdziłowska [2], Magdalena Głowala-Kosińska [2], Wojciech Fidyk [2], Andrzej Smagur [2], Barbara Łasut-Szyszka [3], Agnieszka Gdowicz-Kłosok [3], Iwona Mitrus [2], Marcin Wilkiewicz [2], Agata Chwieduk [2], Daria Burdalska [2], Joanna Korfanty [2], Sebastian Giebel [2-], Marcin Rojkiewicz [1], Andrzej Bak [1], Violetta Kozik [1] *

[1] Institute of Chemistry, University of Silesia, Szkolna 9, 40-007 Katowice, Poland
[2] Department of Bone Marrow Transplantation and Oncohematology, Maria Sklodowska-Curie National Research Institute of Oncology, Gliwice Branch, ul. Wybrzeze Armii Krajowej 15, Gliwice 44-101, Poland
[3] Center for Translational Research and Molecular Biology of Cancer, Maria Skłodowska-Curie National Research Institute of Oncology, Gliwice Branch, ul. Wybrzeze Armii Krajowej 15, Gliwice 44-101, Poland
* Correspondence: Agata.Kawulok@gliwice.nio.gov.pl (A.K.); Violetta.Kozik@us.edu.pl (V.K.)

Table S1. PCCs and BM-MSCs cell lines statistical significance of apoptosis and necrosis after GEM (10 μM), GEM+FLU (10 μM+10 μM) and GEM+FLU (10 μM+2 mM) treatment. The significance ranges used are P ≤ 0.05*, P ≤ 0.01**, P ≤ 0.001***.

**PANC-1**	**Time**	$P_{GEM\ (10\mu M)\ vs.\ GEM+FLU\ (10\mu M+10\mu M)}$	$P_{GEM\ (10\mu M)\ vs.\ GEM+FLU\ (10\mu M+2mM)}$	$P_{GEM+FLU\ (10\mu M+10\mu M)\ vs.\ GEM+FLU\ (10\mu M+2mM)}$
**Viable**	24h	**<0.001**	**<0.001**	**<0.001**
	48h	**<0.001**	**<0.001**	**<0.001**
**Early apoptotic**	24h	0.26	**<0.05**	0.61
	48h	0.16	**<0.001**	**<0.05**
**Late apoptotic/ necrotic**	24h	**<0.001**	**<0.001**	**<0.001**
	48h	**<0.01**	**<0.001**	0.12
**AsPC-1**	**Time**	$P_{GEM\ (10\mu M)\ vs.\ GEM+FLU\ (10\mu M+10\mu M)}$	$P_{GEM\ (10\mu M)\ vs.\ GEM+FLU\ (10\mu M+2mM)}$	$P_{GEM+FLU\ (10\mu M+10\mu M)\ vs.\ GEM+FLU\ (10\mu M+2mM)}$
**Viable**	24h	**<0.001**	**<0.001**	**<0.001**
	48h	**<0.01**	**<0.001**	**<0.001**
**Early apoptotic**	24h	0.14	**<0.001**	**<0.001**
	48h	**<0.001**	**<0.001**	**<0.001**
**Late apoptotic/ necrotic**	24h	**<0.001**	**<0.001**	**<0.01**
	48h	0.49	**<0.01**	**<0.05**
**BxPC-3**	**Time**	$P_{GEM\ (10\mu M)\ vs.\ GEM+FLU\ (10\mu M+10\mu M)}$	$P_{GEM\ (10\mu M)\ vs.\ GEM+FLU\ (10\mu M+2mM)}$	$P_{GEM+FLU\ (10\mu M+10\mu M)\ vs.\ GEM+FLU\ (10\mu M+2mM)}$
**Viable**	24h	**<0.001**	**<0.001**	**<0.01**
	48h	**<0.05**	**<0.001**	**<0.01**
**Early apoptotic**	24h	**<0.05**	**<0.001**	0.32
	48h	0.05	**<0.01**	0.16
**Late apoptotic/ necrotic**	24h	0.34	**<0.01**	**<0.05**
	48h	**<0.001**	**<0.001**	**<0.001**
**BM-MSCs**	**Time**	$P_{GEM\ (10\mu M)\ vs.\ GEM+FLU\ (10\mu M+10\mu M)}$	$P_{GEM\ (10\mu M)\ vs.\ GEM+FLU\ (10\mu M+2mM)}$	$P_{GEM+FLU\ (10\mu M+10\mu M)\ vs.\ GEM+FLU\ (10\mu M+2mM)}$
**Viable**	24h	0.53	**<0.001**	**<0.001**
	48h	0.16	**<0.001**	**<0.001**
**Early apoptotic**	24h	0.46	**<0.001**	**<0.001**
	48h	**<0.001**	0.11	**<0.001**
**Late apoptotic/ necrotic**	24h	0.55	0.99	0.73
	48h	**<0.001**	**<0.001**	**<0.001**

Table S2. PCCs statistical significance of apoptosis and necrosis after CM-GEM and CM-GEM+FLU treatment. The range significances used are $P \le 0.05$*, $P \le 0.01$**, $P \le 0.001$***.

**PANC-1**	**Time**	$P_{\text{Control vs. CM-GEM}}$	$P_{\text{Control vs. CM-GEM+FLU}}$	$P_{\text{CM -GEM vs. CM-GEM+FLU}}$
**Viable**	72h	**<0.001**	**<0.001**	0.06
**Early apoptotic**	72h	**<0.05**	**<0.05**	0.9
**Late apoptotic/ necrotic**	72h	**<0.001**	**<0.001**	**<0.01**

**AsPC-1**	**Time**	$P_{\text{GEM (10μM) vs. GEM+FLU (10μM+10μM)}}$	$P_{\text{GEM (10μM) vs. GEM+FLU (10μM+2mM)}}$	$P_{\text{GEM+FLU (10μM+10μM) vs. GEM+FLU (10μM+2mM)}}$
**Viable**	72h	**<0.001**	**<0.001**	1
**Early apoptotic**	72h	**<0.001**	**<0.001**	0.96
**Late apoptotic/ necrotic**	24h	**<0.001**	**<0.001**	1

**BxPC-3**	**Time**	$P_{\text{GEM (10μM) vs. GEM+FLU (10μM+10μM)}}$	$P_{\text{GEM (10μM) vs. GEM+FLU (10μM+2mM)}}$	$P_{\text{GEM+FLU (10μM+10μM) vs. GEM+FLU (10μM+2mM)}}$
**Viable**	72h	**<0.001**	**<0.001**	0.87
**Early apoptotic**	72h	**<0.001**	**<0.001**	**<0.05**
**Late apoptotic/ necrotic**	24h	**<0.001**	**<0.001**	0.38
